# Recurrent Respiratory Papillomatosis Presenting as Worsening Dyspnea in an HIV-infected Patient

**DOI:** 10.7759/cureus.32908

**Published:** 2022-12-24

**Authors:** Inês C Gonçalves, Carolina Silva, Joana Gomes, Sandra Xará

**Affiliations:** 1 Serviço de Doenças Infeciosas, Centro Hospitalar Universitário do Porto, Porto, PRT; 2 Serviço de Pneumologia, Centro Hospitalar Universitário do Porto, Porto, PRT

**Keywords:** human immunedeficiecy virus (hiv) infection, infectious disease, hpv vaccine, recurrent respiratory papillomatosis, human papillomavirus (hpv)

## Abstract

Recurrent respiratory papillomatosis (RRP) is a rare manifestation of human papillomavirus (HPV) infection. It is characterized by relapsing bulky papillomas in the respiratory tract, which are usually benign in nature. We describe a challenging case of RRP in an HIV-infected patient with extensive pulmonary disease, presenting with worsening dyspnea. The interaction between HPV with HIV as a coinfection is still not completely understood, particularly the role of HIV-associated immunosuppression in RRP.

Our main goal is to raise awareness of this clinical entity and to promote further studies on its management, particularly in specific populations such as HIV-infected individuals. A brief review of the theme is also presented.

## Introduction

Human Papillomavirus (HPV) is a DNA virus of the *Papillomaviridae* family with a propensity to infect epithelial cells, especially in the junction between stratified squamous and respiratory columnar epithelium [1,2]. Recurrent respiratory papillomatosis (RRP) is a rare manifestation of HPV infection, characterized by relapsing exophytic papillomas in the epithelium of the respiratory tract. Its incidence rate is variable, with estimates varying from two to four cases per 100,000 individuals, depending on the age of onset, geographic region, and social status [2,3].

RRP shows a characteristic bimodal age distribution divided into juvenile-onset RRP and adult-onset RRP. Juvenile-onset RRP, occurring in children before the age of 12 years, is usually more aggressive and more likely to recur [2,4]. The adult form usually develops in individuals over 20 years of age, usually in the third and fourth decades of life, is more common in men, and is usually less aggressive with fewer recurrences than in the juvenile form [1,2,4,5]. In juvenile-onset RRP, the virus is thought to be transmitted mainly during childbirth but also during pregnancy, with the presence of maternal anogenital warts (particularly during birth) being the predominant risk factor for this form of the disease [2]. In adult-onset RRP, infection is transmitted through sexual contacts, specifically by oral sexual practices, with the number of sexual partners being suggested as an important risk factor for the development of this form of disease [2].

RRP is difficult to treat given its tendency to recur and spread through the adjacent aerodigestive tract. There is no cure for RRP, and treatment is primarily focused on maintaining airway patency, often requiring multiple endobronchial procedures [2,6]. 

## Case presentation

We describe the case of a 46-year-old female patient with a past medical history of HIV infection with 253 CD4+/mm^3^, without sustained viral suppression due to frequent interruptions of her highly active antiretroviral therapy (HAART) regimen. She also had severe chronic obstructive pulmonary disease (COPD), with bronchiectasis and emphysema, due to lifelong smoking and inhaled drug, as well as a previous episode of tuberculosis. These sequelae resulted in chronic respiratory insufficiency requiring continuous domiciliary oxygen flow at 2L/min, despite optimized long-acting bronchodilator therapy. The patient had not been vaccinated for HPV and previous cervical cytology screening was negative for dysplasia.

**Figure 1:**
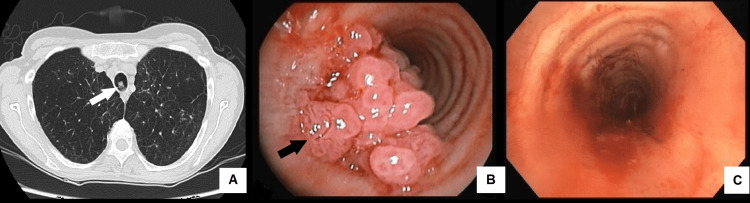
(A) Pulmonary CT scan showing a polypoid lesion (white arrow) on tracheal lumen; (B) Bronchofibroscopy image of an exophytic papilloma (black arrow) obstructing half of trachea's lumen; (C) Rigid bronchoscopy image after mechanical resection of the papilloma with re-establishment of trachea's lumen

She was admitted to the emergency department due to worsening dyspnea requiring increased oxygen flow and productive cough with mucopurulent sputum for over a week, without fever or other accompanying signs or symptoms. An infectious exacerbation of her COPD was assumed. Since there was no improvement after three days of antibiotics (intravenous ceftriaxone 1g/12h and azithromycin 500mg/24h), with negative results in sputum cultures and respiratory virus panel, a pulmonary computed tomography scan was performed that revealed a polypoid lesion on the tracheal lumen (Figure 1A) without any other findings on pulmonary parenchyma beyond previously known sequelae. Fiberoptic bronchoscopy was performed and a biopsy of the papillomatous lesion revealed a squamous cell papilloma without dysplasia with no other pathological findings. Viral genotyping identified low-risk HPV genotype 6. A rigid bronchoscopy was performed, and the papilloma was mechanically excised. The patient was submitted to cryotherapy with posterior argon plasma coagulation on the base of the lesion to prevent relapses. There was a complete resolution of the symptoms. HPV vaccination was proposed but the patient could not afford it due to economical constraints. A follow-up bronchofibroscopy procedure was scheduled in six months but the patient failed to attend.

The patient was re-admitted to our center two years after the first admission with a new episode of worsening dyspnea and stridor. Recurrence of tracheal papillomatosis lesions was documented on a new bronchofibroscopy which revealed obstruction in the lower third of the trachea with a lesion occupying about half of its lumen near the location of the previous papilloma, with no other lesions (Figure 1B). Mechanical resection and laser photocoagulation were performed by rigid bronchoscopy with successful re-establishment of the caliber of the tracheal lumen (Figure 1C). Cauterization of the lesion implantation base was performed to minimize the risk of a new relapse. The patient showed symptomatic improvement after the procedure and returned to her basal oxygen requirements and was stable and asymptomatic in a follow-up appointment four months later. Later on, the patient eventually lost follow-up after subsequent missed appointments and died from unknown causes.

## Discussion

RRP is a disease of the aerodigestive tract that typically affects the larynx but can occasionally become aggressive, resulting in persistent or recurrent involvement of the nasopharynx, tracheobronchial tree, and, more rarely, the pulmonary parenchyma [2]. The sole involvement of the trachea is rather rare [7,8]. The most common symptoms are hoarseness, progressive dyspnea, and stridor as a result of airway obstruction. Less commonly presenting symptoms include chronic cough, recurrent respiratory infections, and acute respiratory distress [2,5].

Even though several distinct genotypes of HPV may cause RRP, current evidence shows that more than 90% of RRP cases are caused by the low-risk HPV genotypes (HPV 6 and 11) with transformation to malignant forms being rare and usually associated with infection by HPV genotype 16 [2,9].

There is no curative treatment for RRP. The mainstay of treatment is endobronchial and surgical debulking, predominantly through laser therapy or the use of microdebriders [3]. Due to the frequent recurrences, multiple procedures may be necessary to maintain airway patency, improve vocal quality, and prevent disease spread and secondary complications [4]. Nonetheless, the recurrence of post-excision papillomas is common and occurs due to the persistence of the viral genome in the spared tissue [2]. Even though high-quality evidence is lacking, adjunctive therapies such as intralesional cidofovir or bevacizumab may be used in cases that require four or more surgical debulkings per year, in cases of rapid recurrences of papilloma with short disease-free intervals and when there is distal airway spread of the disease [4,10]. Programmed death 1 (PD-1) pathway inhibitors are also being investigated as adjunctive therapy, currently under phase two clinical trials [4].

Death secondary to respiratory papillomatosis is rare and most frequently due to pulmonary compromise. Even though dysplasia can be identified in up to 50% of cases with RRP, malignant transformation occurs only in about 1-2% of cases [1].

A preventive strategy through the administration of HPV recombinant vaccines was shown to be effective in reducing the incidence of RRP, particularly juvenile-onset RRP, in a large prospective study developed in Australia following the implementation of a national HPV vaccination program with the quadrivalent HPV vaccine Gardasil (Merck and Co, Inc., Whitehouse Station, USA) which protects against infection caused by HPV types 6, 11, 16, and 18 [11]. The therapeutic effect of HPV vaccines on RRP has also been shown in some studies. The widespread administration of HPV vaccines, both the quadrivalent and the nonavalent Gardasil-9 (Merck and Co, Inc., Whitehouse Station, USA), correlates with an increase in inter-surgical intervals and the decrease in the recurrence of papillomas with higher rates of partial or complete responses [12,13]. The nonavalent HPV vaccine, which protects against HPV genotypes 6, 11, 16, 18, 31, 33, 45, 52 and 58, is the currently adopted vaccine by most countries’ national HPV vaccination programs. It is currently approved and recommended for men and women from ages nine to 45 years for the prevention of anogenital, oropharyngeal, and other head and neck cancers, as well as for the prevention of genital warts, with maximum protection achieved if administered before initiation of sexual activity [14]. Despite encouraging literature on the role of adjuvant HPV vaccination in primary and secondary prevention of RRP, it has not yet been widely accepted and implemented in treating this population, which may be partially due to the lack of additional clinically robust studies and the high costs associated with the vaccine.

Patients with HIV have an increased risk of cervical and anogenital cancers, which is related to the degree of immunosuppression [10,15]. In contrast, there is no established association between HIV and RRP [10]. Nevertheless, HIV infection may increase the risk and aggressiveness of RRP, with some reports associating the presence of HIV with a higher chance for aggressive RRP, particularly in children. Aggressive disease is defined as the presence of extra laryngeal papillomas and/or disease requiring 10 or more surgical debulkings in total [5,15]. Currently, there are no RRP screening guidelines implemented, and physicians should be vigilant and prompt in evaluating the tracheobronchial tree in HIV patients if RRP is suspected, as well as propose HPV vaccination in those unvaccinated, either as primary or secondary prevention.

The presented case intends to show a case of RRP in a patient with a particularly challenging clinical setting due to poorly controlled HIV infection and extensive pulmonary disease. In this case presentation, the symptoms presented by the patient were attributed to an exacerbation of her COPD rendering a delay in diagnosis and appropriate treatment of RRP. This case also shows the recurrent pattern of RRP even after complete excision of the papillomatous lesions. The patient had inconsistent adherence to the health care system, mainly due to her social-economic status and substance abuse; it is unknown if RRP and/or its complications may have been implicated in the cause of death.

## Conclusions

RRP is a chronic and difficult-to-treat disease due to its frequent recurrence rate and unpredictable aggressiveness. The association between HIV and HPV, particularly for RRP, is not fully understood but there seems to be an association between HIV-associated immunosuppression and more frequent and severe manifestations of HPV infections, namely RRP. Therefore, it seems reasonable to think that HPV vaccination should be recommended for all unvaccinated patients who present with RRP, particularly for HIV-infected patients, regardless of age at presentation, and that it should be included in national vaccination programs for this population. The presented case intends to show the difficulties in the management of RRP and the need for frequent revaluations and endobronchial/surgical procedures.
